# CoV-RBD121-NP Vaccine Candidate Protects against Symptomatic Disease following SARS-CoV-2 Challenge in K18-hACE2 Mice and Induces Protective Responses That Prevent COVID-19-Associated Immunopathology

**DOI:** 10.3390/vaccines9111346

**Published:** 2021-11-17

**Authors:** Jennifer K. DeMarco, Joshua M. Royal, William E. Severson, Jon D. Gabbard, Steve Hume, Josh Morton, Kelsi Swope, Carrie A. Simpson, John W. Shepherd, Barry Bratcher, Kenneth E. Palmer, Gregory P. Pogue

**Affiliations:** 1 Center for Predictive Medicine for Biodefense and Emerging Infectious Diseases, University of Louisville, Louisville, KY 40202, USA; jennifer.wolf.2@louisville.edu (J.K.D.); william.severson@louisville.edu (W.E.S.); jon.gabbard@louisville.edu (J.D.G.); kenneth.palmer@louisville.edu (K.E.P.); 2 Department of Microbiology and Immunology, University of Louisville, Louisville, KY 40202, USA; 3 Kentucky BioProcessing, Inc., Owensboro, KY 42301, USA; humes@kentuckybioprocessing.com (S.H.); mortonj2@kentuckybioprocessing.com (J.M.); swopek@kentuckybioprocessing.com (K.S.); simpsoc3@kentuckybioprocessing.com (C.A.S.); shephej@KentuckyBioProcessing.com (J.W.S.); bratchb1@rjrt.com (B.B.); gppogue@ymail.com (G.P.P.); 4 James Brown Cancer Center, University of Louisville, Louisville, KY 40202, USA; 5 Department of Pharmacology and Toxicology, University of Louisville, Louisville, KY 40202, USA; 6 IC² Institute, University of Texas at Austin, Austin, TX 78805, USA

**Keywords:** SARS-CoV-2, COVID-19, betacoronavirus, vaccine, betacoronaviridae, TMV

## Abstract

We developed a SARS-CoV-2 vaccine candidate (CoV-RBD121-NP) comprised of a tobacco mosaic virus-like nanoparticle conjugated to the receptor-binding domain of the spike glycoprotein of SARS-CoV-2 fused to a human IgG1 Fc domain. CoV-RBD121-NP elicits strong antibody responses in C57BL/6 mice and is stable for up to 12 months at 2–8 or 22–28 °C. Here, we showed that this vaccine induces a strong neutralizing antibody response in K18-hACE2 mice. Furthermore, we demonstrated that immunization protects mice from virus-associated mortality and symptomatic disease. Our data indicated that a sufficient pre-existing pool of neutralizing antibodies is required to restrict SARS-CoV-2 replication upon exposure and prevent induction of inflammatory mediators associated with severe disease. Finally, we identified a potential role for CXCL5 as a protective cytokine in SARS-CoV-2 infection. Our results suggested that disruption of the CXCL5 and CXCL1/2 axis may be important early components of the inflammatory dysregulation that is characteristic of severe cases of COVID-19.

## 1. Introduction

Since its appearance in December 2019, SARS-CoV-2 has spread to more than 180 countries, infecting more than 214 million people with over 4.4 million deaths as of August 2021 [1]. To date, three betacoronaviruses capable of causing severe disease and mortality in humans have been identified: severe acute respiratory syndrome (SARS-CoV), Middle East respiratory syndrome (MERS-CoV), and the newly identified coronavirus (CoV) SARS-CoV-2 [2,3,4], which is the causative agent of Corona Virus Disease 19 (COVID-19) [5].

Shortly after its emergence in Wuhan, China, SARS-CoV-2 was rapidly identified by Zhu et al. [4] as a zoonotic betacoronavirus closely related to SARS-CoV and MERS-CoV that arose from a recombination event between the bat and pangolin clades [6,7]. Similar to other members of the betacoronaviridae, SARS-CoV-2 has a large genome of approximately 30 kilobase pairs (Kbp) surrounded by an envelope comprised of S (spike), N (nucleocapsid), E (envelope), and M (membrane) proteins [7]. Entry into host cells is achieved through a receptor-binding domain (RBD) on the viral S protein. The RBD is part of the S1 domain. The RBD binds to the human angiotensin II converting enzyme receptor (hACE2) [7]. Following binding to hACE2, S protein is cleaved at a site between the S1 and S2 domains, resulting in a conformational change that facilitates viral fusion [7,8].

Despite ongoing efforts, options for successful intervention after infection with SARS-CoV-2 remain of limited usefulness. In contrast, vaccination is a proven strategy to reduce both the severity and frequency of infection, and vaccines targeting the S protein of SARS-CoV-2 have proven effective in significantly reducing morbidity and mortality [9,10]. However, the continued emergence of variants and increasing severity of resulting economic crises following multiple waves of the pandemic has led to an unprecedented race to develop and deliver new and effective vaccine platforms that can be rapidly adapted to virus mutations and that produce vaccines that can be rapidly manufactured and distributed worldwide.

Of the vaccine platforms employed in the pandemic to date, distribution and stability remain a challenge because many third-world countries lack the infrastructure to maintain the extreme cold-chain requirements to successfully transport and store mRNA-based vaccines [11]. In addition, the appearance of an abbreviated approval process and documented adverse side effects with the current spectrum of available EUA-approved vaccines have created a significant degree of vaccine hesitancy in many regions [12,13,14]. This comes at a time when emerging SARS-CoV-2 variants have changes in the S protein that reduce the effectiveness of vaccines developed against the original Wuhan isolate [15]. In the last two months, the highly transmissible Delta variant has become the dominant strain of SARS-CoV-2 in most of the world [16,17,18] and has been increasingly associated with breakthrough infections in vaccinated individuals [16,19]. The frequency of breakthrough infections now documented worldwide and reduced efficacy of the currently approved vaccines in eliciting cross-protective antibodies against the now dominant Delta variant have created a need to understand the relationship between antibody titers and prevention of symptomatic disease upon infection with this virus. Additionally, more insights are needed into the downstream impact of vaccination on inflammatory cytokine production that determine the outcome of infection in COVID-19.

Plant-based nanoparticle (NP) vaccines are highly antigenic, can be readily designed in a multi-valent format against specific targets, and are often associated with minimal side effects in vivo [20,21]. Moreover, they have been effective in elderly populations during Phase III influenza efficacy trials [22]. Plant-based viral expression systems can be used to elicit antibody production against selected epitopes thought to be important in neutralization of selected pathogens. In a companion study, we reported the development of a novel NP vaccine platform incorporating the SARS-CoV-2 receptor-binding domain (RBD) that is safe and highly immunogenic [23]. Furthermore, the formulations of this vaccine, CoV-RBD121-NP, are stable for up to 12 months at 2–8 or 22–28 °C [23].

In this report, we evaluated the efficacy of CoV-RBD121-NP in eliciting strong antibody responses in K18-hACE2 mice and providing protection from SARS-CoV-2 challenge in vivo. K18-hACE2 mice are genetically engineered to express human ACE2 in epithelial cells of the airway and other organs and represent a preclinical animal model of COVID-19 [24,25]. In addition, we compared IgG responses from immunized mice with those from recovered human COVID-19 patients to understand the relationship between neutralizing antibody titers, geometric mean titer (GMT), and seroconversion to specific IgG isotypes in protection against SARS-CoV-2 infection. By examining sera collected at two times after infection from mice challenged with SARS-CoV-2, we identified two distinct phases of inflammatory dysregulation and a possible role for C-X-C motif chemokine ligand 5 (CXCL5) as a key modulator of the early stages of inflammatory dysregulation that leads to mortality in COVID-19. Finally, we characterized the relationship of pre-infection neutralizing antibody levels with prevention of symptomatic disease in SARS-CoV-2 infection and showed that a two-dose course of CoV-RBD121-NP provides complete protection from virus-induced disease, pathology, and mortality in a humanized mouse model of SARS-CoV-2.

## 2. Materials and Methods

### 2.1. Vaccination of Mice and SARS-CoV-2 Challenge

Six-week-old female K18-hACE2 (Jackson Laboratories, Bar Harbor, ME, USA) mice were immunized with either CoV-RBD121-NP without adjuvant (15 or 45 µg, *n* = 10 per group) or vehicle [(phosphate-buffered saline (PBS)] control (*n* = 7) on day 0 by intramuscular injection in a total volume of 100 µL. Twenty-one days later, blood samples were collected from each animal by cheek bleed and processed in serum separator tubes by centrifugation at 1000× *g* for 5 min at room temperature. Mice were then given a second dose of vaccine (15 or 45 µg) via intramuscular injection. Blood was drawn day 36 (post-initial vaccination) and processed to collect serum and evaluate antibody response prior to challenge. Mice were then challenged with 1 × 10^5^ pfu in 50 µL of USA-WA1/2020 SARS-CoV-2 (2019-nCoV) by intranasal delivery forty-two days after initial vaccination with either CoV-RBD121-NP or vehicle control. Mice were evaluated twice daily for clinical symptoms and weight loss for 10 days post-challenge. Categories included in clinical scoring were (i) posture and appearance of fur (piloerection) (0–3) and (ii) development of respiratory distress (0–3). Four days following challenge, blood samples were collected from mice by cheek bleed and pooled per group. Blood samples were collected from all mice again at the conclusion of this study (day 10 post-infection) or when the animal reached end point criteria. All vaccine materials were stored at 4 °C for a duration of less than two months prior to use. All mouse work was approved by the University of Louisville Institutional Animal Care and Use Committee (University of Louisville, IACUC 19625, approved 9 September 2021), and all procedures were performed in the university’s certified animal biosafety level three laboratory.

### 2.2. Human Convalescent Sera

In mid-March of 2020, prior to availability of EUA-authorized vaccines, health care workers who had actively been in contact with SARS-CoV-2 patients were invited to participate in a study to examine infection rates and immune responses to SARS-CoV-2 [26]. Our analysis of the sera in this study was approved by the Institutional Review Board at University of Louisville (IRB Approval #20.0312).

### 2.3. Neutralizing Titer Assay

Vero E6 cells (ATCC, cat# VERO C1008; CRL-1586, Manassas, VA, USA) were seeded at a density of 2 × 10^4^ cells in 96-well tissue culture plates (Corning/Costar) and incubated overnight at 37 °C with 5% CO_2_. The following morning, cells were washed once with Vero incubation medium [VIM: 100 µL DMEM containing penicillin/streptomycin and 5% fetal bovine serum (FBS)] followed by two washes with 200 µL of PBS prior to the addition of 100 µL of VIM. Prior to the addition of virus, 2-fold serial dilutions of serum samples were prepared in VIM in a separate 96-well dilution plate. SARS-CoV-2 was then added to the dilution plate at a concentration of 60 pfu/well for a final multiplicity of infection (MOI) of 0.003 and incubated with the serum at 37 °C, 5% CO_2_. Following a 1 h incubation, medium on the cells was replaced with 100 µL of the serum and virus mixture, and the cells were returned to the incubator. After four days, cells were fixed with 4% paraformaldehyde and stained with 1% crystal violet for 20 min at room temperature. Cells were then washed twice with 200 µL of filtered tap water and assessed for cytopathic effects (CPE).

For assessment of neutralization titer in samples from mice collected at day 4 post-challenge, the sera were pooled for evaluation due to limited volume of blood that could be collected without affecting the health of the mouse. Neutralizing titers were evaluated with four technical replicates per group of pooled sera. For neutralization titer assessment at baseline, post-prime (day 21), post-boost (day 36), and end point, serum from each mouse was evaluated separately.

### 2.4. RBD-Directed Antibody ELISA

For analysis of sera from mice, RBD-specific anti-IgG titers were measured by ELISA against recombinant RBD domain of the SARS-CoV-2 S protein (SARS-CoV-2/Wuhan/2019 RBD-6xHis-Human Fc, Immunetech, New York, NY, USA, IT-002-031p). Briefly, 96-well plates (Nunc Maxisorb, ThermoFisher Scientific, Waltham, MA, USA) were coated with 2.5 µg/mL RBD-Fc in 100 µL of PBS overnight at 4 °C. Plates were then washed three times with 200 µL/well of PBS + 0.2% Tween 20 prior to the addition of 200 µL/well of protein-free blocking solution (Pierce Protein Free Blocking Buffer, Thermo Scientific #37572). Plates were incubated with the blocking solution for 2 h at room temperature and then washed twice with 200 µL of PBS + 0.2% Tween 20. Serum samples diluted in protein-free blocking buffer were then added to the plate in a final volume of 100 µL/well and incubated at room temperature for 1 h. Following incubation, the plates were washed with 200 µL of PBS + 0.2% Tween 20 per well three times prior to the addition of an anti-IgG-conjugated detection antibody as appropriate at a dilution of 1:5000 in protein-free blocking buffer (goat anti-mouse IgG1-HRP, Southern Biotech, Birmingham, AL, USA, cat#1030-05; goat anti-mouse IgG (H + L) HRP, Southern Biotech #1036-05; goat anti-mouse IgG2a HRP, Southern Biotech #1081-05; goat anti-mouse IgG2c HRP, Southern Biotech #1078-05; goat anti-mouse IgG1 HRP, Southern Biotech #1071-05). The detection antibody was incubated for 1 h at room temperature. Following incubation, the plates were washed with 200 µL of PBS + 0.2% Tween 20 per well three times prior to the addition of 100 µL of TMB (3,3′, 5,5′-tetramethylbenzidine). Plates were allowed to develop for 10 min at room temperature and read at 450 nm on a Synergy plate reader (Biotek, Winooski, VT, USA). Background values were calculated from three blank control wells and subtracted prior to the determination of a cutoff threshold based on the average negative control value (non-specific IgG followed by the detection antibody), which was then used to determine the last dilution with detectable antibodies.

For evaluation of mouse samples, sera were pooled and three or four technical replicates for each of the pooled samples were assayed. GMT values were compiled in GraphPad Prism (9.0.0), San Diego, CA, USA and assessed for statistical significance compared with vehicle controls (ANOVA, Tukey’s post hoc test).

For analysis of sera from human convalescent COVID-19 patients, quantities were limited. To evaluate human IgG1, IgG2, IgG3, and IgG4 responses, we used the methods described by Hamorsky et al. [26]. Briefly, RBD-specific anti-IgG titers were measured by ELISA against recombinant RBD domain of the SARS-CoV-2 S protein (SARS-CoV-2/Wuhan/2019 RBD-His, Immunetech, New York, NY, USA, IT-002-036p). There were insufficient sera available to perform a full dilution series with sufficient replicates to generate GMT titers for multiple isotypes. We therefore evaluated the lowest dilution only (1:100) for all four isotypes and directly compared the data from the ELISA (presented as absorbance). The following antibodies were used to detect human IgG isoforms: goat anti-human IgG (H + L)-HRP, Southern Biotech #2015-05; mouse anti-human IgG1-HRP, Southern Biotech #9052-05; mouse anti-human IgG4-HRP, Southern Biotech #9190-05; mouse anti-human IgG3-HRP, Southern Biotech #9210-05; mouse anti-human IgG2-HRP, Southern Biotech #9060-05.

### 2.5. Histopathology and Immunohistochemistry

Lung tissues collected from euthanized mice were fixed in 10% formalin for 48 h at room temperature and processed into paraffin-embedded blocks for sectioning. Slides were prepared, stained with hematoxylin/eosin, and evaluated by an accredited veterinary laboratory (LADDL, Louisiana State University). For immunohistochemistry, de-paraffinized sections of lung tissue were covered with 100 µL with trypsin (0.05%) in water with 0.1% calcium chloride (pH 7.8) for 20 min at 37 °C. Sections were then rinsed with tap water and blocked with 5% skim milk in PBS at room temperature for one hour. After blocking, slides were washed twice with PBS + 0.1% Triton X-100. Sections were then incubated with a 1:250 dilution of detection antibody in PBS + 1% skim milk for 1 h at room temperature. Detection antibodies were FITC-Ly6G (ThermoFisher, Waltham, MA, USA cat# 11-9668-82), APC-Cy7-CD14 (BD Pharmingen cat# 560636), CD163 (Abcam cat# 182422), and FITC-CD11b (BioLegend, San Diego, CA, USA, cat#101205). Following incubation with the detection antibodies, slides were washed three times with PBS + 0.1% Triton X-100. For CD14+CD11b+CD163+ staining only, slides were then incubated for one hour at room temperature with a 1:500 dilution of anti-rabbit IgG-AlexaFluor 555 in PBS + 1% skim milk. Slides were then washed three times with PBS + 0.1% Triton X-100. All slides were then blotted dry, sealed with mounting medium containing DAPI (ThermoFisher Scientific, Waltham, MA, USA, cat#P36935), and allowed to dry overnight before imaging on a LSM 7 confocal microscope (Zeiss). Multiple images (14 to 35) per group distributed evenly across individuals were assessed for each set of markers evaluated. Image analysis was performed in Image J and assessed with GraphPad Prism 9.0.0 to identify statistically significant differences between animal groups.

### 2.6. TCID50 Assays

Lungs and nasal turbinates were homogenized in 400 µL sterile PBS (VWR, Radnor, PA, USA) in a 2-mL tube containing ceramic homogenizing beads (VWR, Radnor, PA, USA, cat#10158-610). To evaluate viral titers, Vero E6 cells (ATCC, Manassas, VA, USA #CRL-1586) grown in DMEM (VWR 16777-200) with penicillin/streptomycin (P/S, VWR 97062-806) and 10% FBS (GIBCO, Waltham, MA, USA, cat# 16000-044) were seeded into a 96-well plate at a density of 2 × 10^4^ cells/well the night prior to the assay. Cells were washed with PBS and resuspended in DMEM + P/S + 5% FBS. Serial dilutions of each tissue homogenate were added to the wells and the plate was incubated at 37 °C, 5% CO_2_. After 3 days, cells were fixed with 10% formaldehyde, and stained with 0.1% crystal violet to visualize CPE. Wells were scored for CPE and the resulting scores were used to calculate the TCID50/g, corrected for the weight of each lung homogenate or nasal turbinate sample.

### 2.7. Cytokine Analysis

Sera were prepared from blood samples taken at four days post-challenge and from samples collected following euthanasia. Serum samples collected four days post-challenge were pooled for each group prior to analysis. Lungs from K18-hACE2 mice infected with SARS-CoV-2 were collected following euthanasia, weighed, and homogenized in 400 µL of PBS by vortexing for 5 min 2 mL tissue homogenizer tubes with ceramic beads (VWR 10158-612). Following homogenization, 50 µL of each serum sample or homogenate was evaluated in duplicate using the Mouse Cytokine and Chemokine 36-Plex Procarta 1A Panel (ThermoFisher Scientific, Waltham, MA, USA, cat#EPXR360-26092-901) per the manufacturer’s instructions. Data were collected using a FlexMap3D instrument (Luminex, Austin, TX, USA) and exported to Microsoft Excel (Microsoft, Redmond, WA, USA) for analysis with GraphPad Prism (San Diego, CA, USA). Concentrations (pg/mL) of each cytokine and chemokine were interpolated from a standard curve following the kit manufacturer’s instructions.

### 2.8. Statistical Analysis

All statistical analysis were performed in GraphPad Prism (version 9.0.0, San Diego, CA, USA). Data were evaluated by two-way ANOVA with Tukey’s post hoc test, two-way ANOVA with Kruskal-Wallis test, or by one-way ANOVA with Fisher’s least significant difference (LSD) post hoc test, where appropriate. Survival data were evaluated with log-rank test.

## 3. Results

### 3.1. K18-hACE2 Mice Exhibit an Antibody Response to CoV-RBD121-NP Vaccination That Resembles the Response in Serum from Convalescent Human COVID-19 Patients

**Figure 1:**
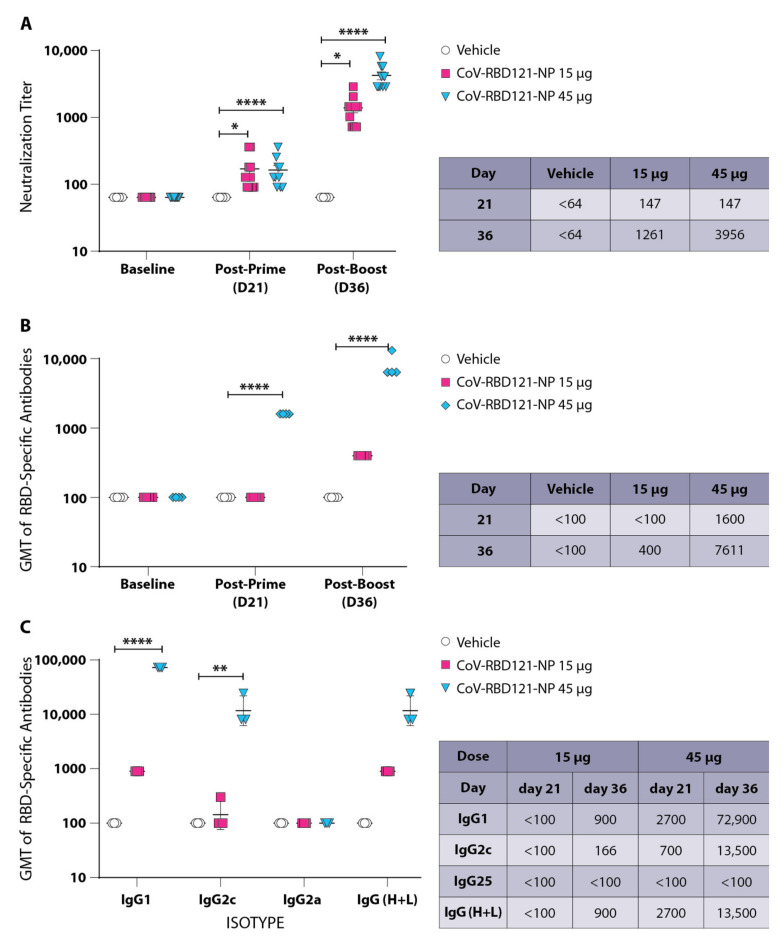
Immune response of K18-hACE2 mice following prime and boost vaccination with CoV-RBD121-NP. (**A**) Graph shows neutralization titer of each mouse in each group at baseline, 21 days after the first dose [post-prime (D21)], and after the second dose [post-boost (D36)] from blood collected 36 days after the first dose. Vehicle, 7 mice; 15 µg CoV-RBD121-NP, 10 mice; 45 µg CoV-RBD121-NP, 10 mice. (**B**) Pooled sera from in each group were evaluated for the presence of RBD-specific IgG antibodies by ELISA at the indicated times. Data are presented for technical replicates of the pooled sera. (**C**) Isotypes of IgG antibodies were detected in pooled sera analyzed in triplicate from mice in the indicated groups. Graph shows results for day 36 sera after initial dose and receipt of boost (post-boost). Chart shows GMT of each isotype from pooled sera from day 21 (post-prime) or day 36 (post-boost). Data are presented for the technical replicates. Significance was determined by two-way ANOVA with Tukey’s post hoc test: * *p* < 0.05; ** *p* < 0.01; **** *p* < 0.0001.

Immunization of K18-hACE2 mice with a single dose (prime) of CoV-RBD121-NP induced a neutralizing antibody response after 21 days in both the 15 µg (*p* = 0.0341) and 45 µg (*p* < 0.0001) groups compared to the vehicle control (Figure 1A). Administration of a second dose (boost) resulted in an 8.58-fold and 26.91-fold increase in the 15 and 45 µg groups, respectively. The average neutralization titer was less (1261) for mice that received 2 doses of CoV-RBD121-NP at 15 µg than for the mice that received the 45 µg doses (3946) (Figure 1A). No adverse effects were noted following administration of CoV-RBD121-NP at either 15 or 45 µg during the course of this study as measured by behavior, weight gain and feeding habits (unpublished data).

To evaluate the IgG antibodies directed against the RBD, we assayed pooled sera from mice in each group. We detected robust induction of IgG after a single dose in the 45 µg group (Figure 1B), which increased 4.76-fold following a second dose to 7611 (*p* < 0.0001) compared to vehicle alone, suggesting that 45 µg induces a strong antibody response with efficient seroconversion to IgG. Pooled sera collected from the 15 µg group exhibited less efficient seroconversion to IgG with no detectable IgG following the first dose of vaccine (Figure 1B), despite induction of neutralizing antibodies following the initial dose (Figure 1A).

Isotyping of the IgG response revealed a dominant IgG1 response with moderate induction of IgG2c after the first dose in the 45 µg CoV-RBD121-NP group (Figure 1C, *p* < 0.0001 for IgG1 and *p* = 0.0105 for IgG2c compared to vehicle). By 36 days after the initial vaccination, the second dose of 45 µg increased both IgG1 and IgG2c levels by ~81-fold over that detected at day 21 after the first dose (Figure 1C, chart). Neither the first nor second dose of either quantity of CoV-RBD121-NP resulted in seroconversion to IgG2a.

**Figure 2:**
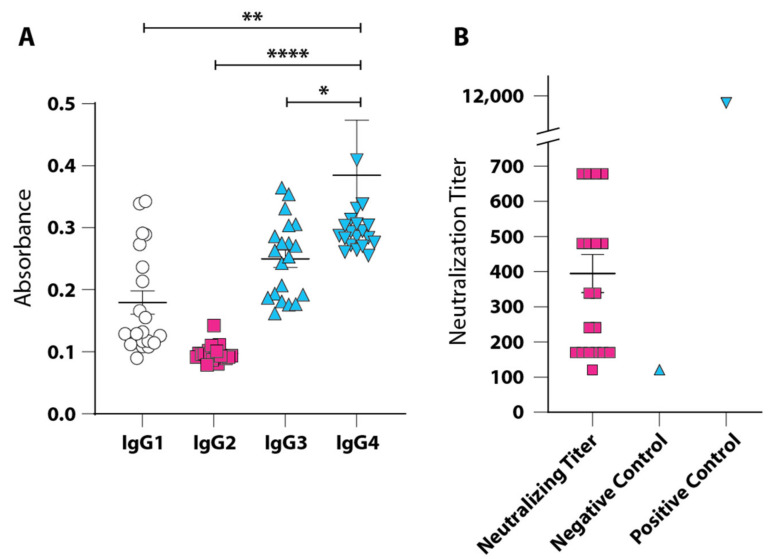
IgG isotypes in serum from unvaccinated, convalescent COVID-19 patients. (**A**) Evaluation of the sera for IgG isotypes of RBD-directed antibodies in 20 convalescent patients. Data are presented as ELISA absorbance values due to limited sample volume. (**B**) Neutralization titers in the serum samples ranged from 170 to 960 (GMT = 328). Serum from a naïve donor who had not been vaccinated or infected served as the negative control and serum from a donor who had received two doses of the Pfizer/BioNTech vaccine as the positive control. Statistical differences were calculated with one-way ANOVA with Fisher’s LSD: * *p* <0.05; ** *p* < 0.01; **** *p* < 0.0001.

To determine whether the humoral immune response in human survivors of COVID-19 correlates with that observed in vaccinated K18-hACE2 mice, we analyzed the IgG isotypes in sera obtained from 20 unvaccinated, convalescent COVID-19 patients [26]. We found a dominant IgG4 response that was significantly increased compared with IgG1 (*p* = 0.0022), IgG2 (*p* < 0.0001), or IgG3 (*p* = 0.041) (Figure 2A), with a rank order of titers as IgG4 > IgG3 > IgG1 > IgG2. This correlates with a dominant IgG1 response observed in the mouse model, because both human IgG4 and mouse IgG1 are functionally equivalent and do not interact with C1q. Neutralization titers ranged from 170 to 960 with a GMT of 328 (Figure 2B).

### 3.2. K18-hACE2 Mice Are Protected from SARS-CoV-2-Induced Disease and Mortality by CoV-RBD121-NP Vaccination

**Figure 3:**
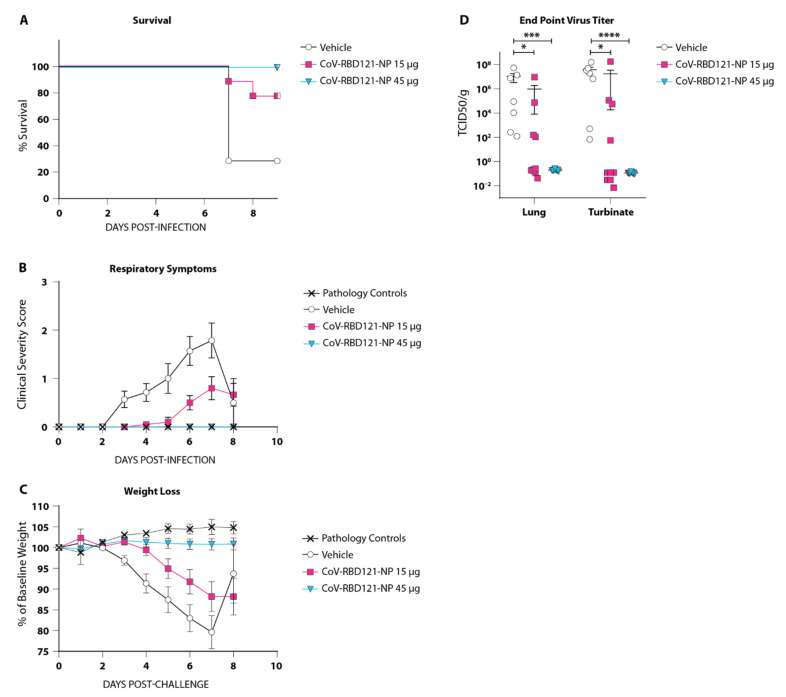
Survival, symptoms, and viral burden in mice vaccinated with CoV-RBD121-NP and challenged with SARS-CoV-2. (**A**) Survival of K18-hACE2 mice following challenge with SARS-CoV-2 (1 × 10^5^ pfu administered intranasally). Compared to the mice receiving vehicle, survival was significantly increased for the group receiving 2 doses of 15 µg of the vaccine (*p* = 0.0420) and for the group receiving 2 doses of 45 µg of the vaccine (*p* = 0.002) by log-rank test; vehicle, n = 7; 15 µg CoV-RBD121-NP group, *n* = 10; 45 µg CoV-RBD121-NP group (*n* = 10). (**B**) Respiratory symptoms following SARS-CoV-2 challenge were assessed on a scale of 0–3 and plotted as the mean ± SEM for each group. Pathology controls are mice that were not challenged with SARS-CoV-2 (*n* = 3). Compared to mice receiving vehicle, symptoms were significantly reduced on day 6 and 7 post-infection for mice receiving 45 µg CoV-RBD121-NP (*p* < 0.0001 for both days) or 15 µg CoV-RBD121-NP (*p* = 0.0062 for day 6 and *p* = 0.0146 for day 7). (**C**) Weight of mice in the indicated groups was measured and plotted as the mean ± SEM percent reduction from baseline for each group. (**D**) Viral titers were assessed on day 10 after challenge or upon death from lungs and nasal turbinates of mice in each group and are plotted as the median tissue culture infectious dose (TICD50) per gram of tissue. Compared to mice receiving vehicle, viral titers were significantly reduced in the lungs (*p* = 0.0005) and nasal turbinates (*p* = 0.0008) from mice in the 45 µg CoV-RBD121-NP group. Statistical differences were calculated with one-way ANOVA with Tukey’s post hoc test in B and C and with two-way ANOVA in D with Kruskal–Wallis test, * *p* < 0.05; *** *p* < 0.001; **** *p* < 0.0001. Key for A and D is presented to the right of D; key for B and C is presented to the right of C.

To evaluate the efficacy of CoV-RBD121-NP in the prevention of COVID-19, the vaccinated mice were challenged intranasally with a lethal dose of SARS-CoV-2 (hCoV-19/USA-WA1/2020) forty-two days following initial vaccination. Vaccination with the two doses of 15 µg of CoV-RBD121-NP significantly reduced mortality from 70% in the vehicle group to 20% in the vaccinated group (*p* = 0.0420), and two doses of 45 µg CoV-RBD121-NP completely prevented mortality (*p* = 0.0020) (Figure 3A). A dose-dependent reduction in the severity of respiratory symptoms was observed with complete protection from respiratory symptoms observed in mice receiving 45 µg CoV-RBD121-NP and a significant reduction symptom severity in the 15 µg group (*p* = 0.0062 on day 6 and *p* = 0.0146 on day 7 compared to mice receiving vehicle). Mice that received 45 µg CoV-RBD121-NP maintained body weight after SARS-CoV-2 challenge (Figure 3B).

We measured viral titers on day 10 (experiment end) or upon death after SARS-CoV-2 challenge in the lungs or nasal turbinates for each group. Vaccination with 45 µg of CoV-RBD121-NP resulted in complete suppression of viral replication in the lungs and nasal turbinates compared to the vehicle control (Figure 3D). In contrast, incomplete protection was observed in the 15 µg group, with two animals harboring low levels of SARS-CoV-2 in the lungs and three animals harboring the virus in nasal turbinates. No detectable live virus was present in the lungs at ten days following challenge in any of the eight surviving animals in the 15 µg group. None of the mice in the 45 µg group had live virus in either the lungs or nasal turbinates at the conclusion of this study.

**Figure 4:**
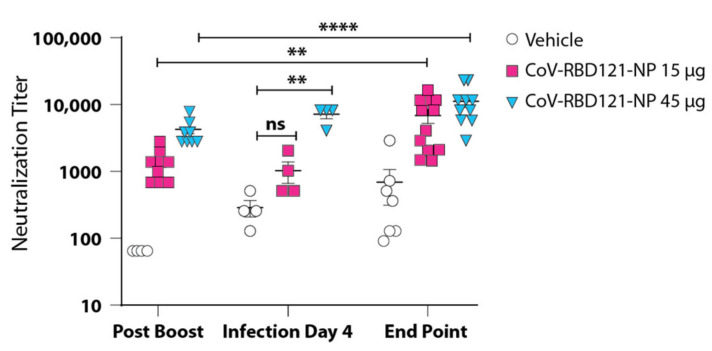
Neutralizing antibody titers in vaccinated mice after challenge with SARS-CoV-2. Titers of neutralizing antibodies were analyzed from serum collected from mice in each group at 4 days following challenge (Infection Day 4, *n* = 4 technical replicates of pooled sera from all mice in each group) or at day 10 or at death (End Point, *n* = 7 for vehicle, *n* = 10 for each vaccine group) following challenge. Post-boost values are the same as those shown in Figure 1A for ease of comparison. Data are plotted as mean ± SEM with values for each mouse shown. Significance was determined by one-way ANOVA: ** *p* < 0.01; **** *p* < 0.0001, ns = not significant.

To evaluate if differences in the titer of neutralizing antibodies in mice that received the 15 or 45 µg doses of the vaccine corresponded to the differences in symptoms, viral load, and lethality after challenge with SARS-CoV-2, we evaluated neutralizing antibody titers 4 days following challenge and in surviving mice at the end of the experiment 10 days following challenge. The neutralization titer in the 45 µg group was significantly higher than that in the 15 µg CoV-RBD121-NP group (*p* = 0.0002), with the level of neutralizing antibodies in the 15 µg group increasing to comparable levels prior to the conclusion of this study (Figure 4). Two of the mice in the 15 µg group continued to harbor low level virus titers in both the lungs and nasal turbinates even after complete recovery from symptomatic disease at ten days post-challenge despite having comparable neutralization tiers to the 45 µg group at the conclusion of this study.

We also compared the neutralization titers before challenge and on day 4 after challenge (Figure 4). Only the mice that received the higher dose had a significant increase in neutralizing antibody titers 4 days after challenge with SARS-CoV-2. Our data suggested that that the presence of sufficient neutralizing antibodies at the onset of infection is important in preventing persistent infection with SARS-CoV-2.

**Figure 5:**
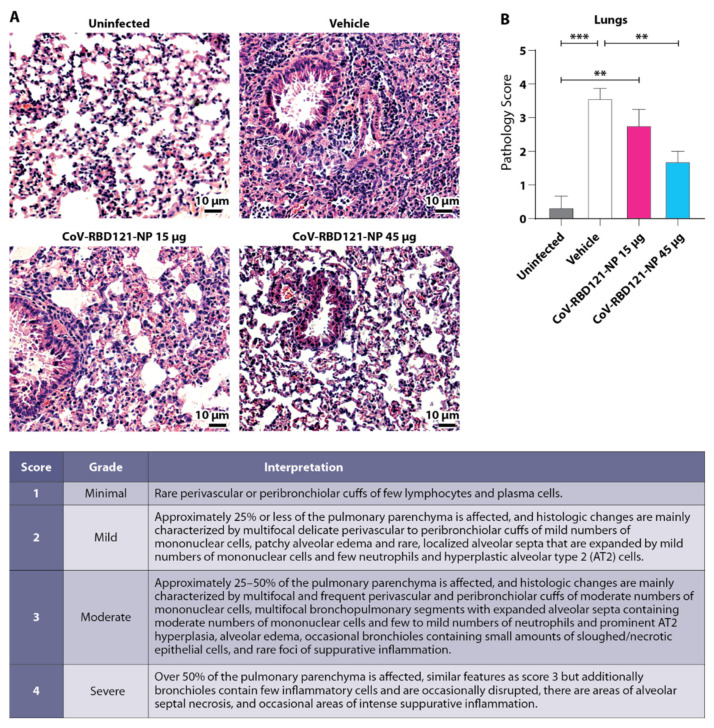
Lung pathology in lungs of mice vaccinated with CoV-RBD121-NP and challenged with SARS-CoV-2. (**A**) Lung tissue was collected upon death or at the end of the experiment from mice in each group, as well as from age- and sex-matched mice that were not challenged with SARS-CoV-2 (uninfected, *n* = 3). Representative tissue sections stained with hematoxylin and eosin are shown. Scale bar = 10 µm. (**B**) Pathology was quantified using the scoring criteria (chart) from 3 to 10 images for each group. Statistical differences were determined by one-way ANOVA with Tukey’s post hoc test, ** *p* < 0.01, *** *p* < 0.001.

Histopathological assessment of the lungs in the mice was scored on a scale of 1 to 4, with 4 representing the most severe pathology and 0 representing histology typical of healthy tissue (Figure 5). Uninfected mice had little evidence of pathology, whereas mice in the vehicle group had frequent multi-focal perivascular and peribronchiolar infiltration of mononuclear cells and neutrophils, alongside prominent alveolar type 2 cells hyperplasia, alveolar edema, and necrosis of epithelial cells affecting 25–50% or more of the pulmonary parenchyma with an average severity score of 3.6 (Figure 5A,B). Vaccination with 45 µg of CoV-RBD121-NP significantly reduced damage to the lungs compared to vehicle alone with an average severity score of 1.7 (*p* = 0.0073 compared to vehicle) (Figure 5B). These mice had notably reduced mononuclear cell infiltration and alveolar edema (Figure 5A). No significant differences in pathology between either the 15 µg group and the vehicle control or the 15 µg group and the 45 µg group were found.

### 3.3. Either Dosing Regimen of CoV-RBD121-NP Vaccination Reduces Immune Cell Accumulation in K18-hACE2 Mice Challenged with SARS-CoV-2

**Figure 6:**
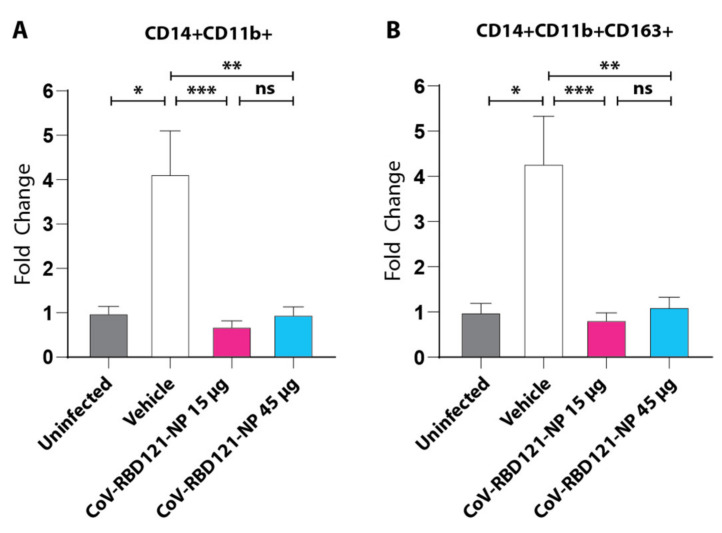
Effect of vaccination on monocytes and macrophages infiltration into lung tissue following challenge with SARS-CoV-2. (**A**,**B**) Lung tissues were stained with antibodies to identify monocytes (CD14+CD11b+) cells (**A**) or macrophages (CD14+CD11b+CD163+). Data from quantification of 14–35 images per group are plotted relative to those in tissue from uninfected mice with significance determined by one-way ANOVA with Tukey’s post hoc test, * *p* < 0.05, ** *p* < 0.01, *** *p* < 0.001, ns = not significant.

To elucidate the immune cell populations associated with inflammatory infiltrates and tissue damage in the mice in the vehicle group and determine whether vaccination reduces recruitment of these cells to the lungs in this model, we stained sections of lung tissue for the presence of monocytes (CD14+CD11b+) and monocyte-derived macrophages (CD14+CD11b+CD163+) and evaluated these samples via confocal microscopy (Figure 6). 

Consistent with previous studies [24,27,28], we observed a significant increase in recruitment of these cells to the lungs in mice in the vehicle group (*p* = 0.0202 for monocytes and *p* = 0.0255 for macrophages) compared with uninfected controls. Mice that received either dose (prime and boost) of the vaccine had significantly reduced infiltration of both monocytes and macrophages to amounts similar to those in the uninfected mice.

**Figure 7:**
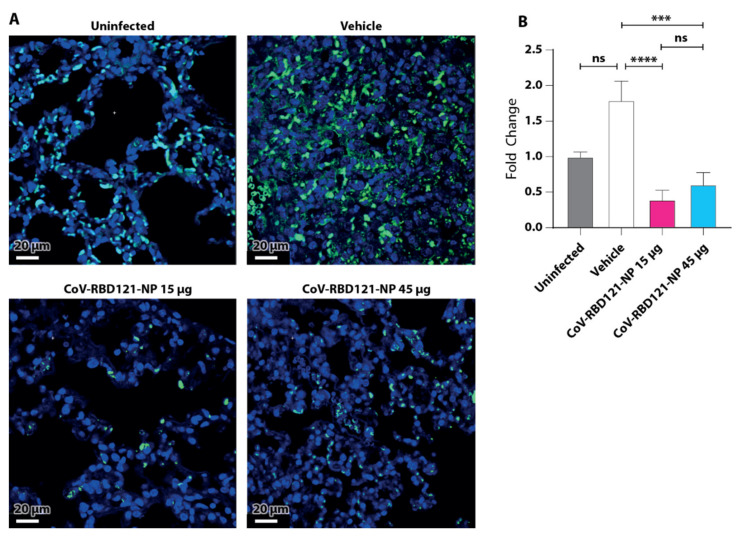
Effect of vaccination on neutrophil infiltration into lung tissue following challenge with SARS-CoV-2. Lung tissues were stained with antibodies to Ly6G to identify neutrophils. (**A**) Representative images are shown for each group. Scale bar = 20 µm. (**B**) Data from quantification of 15–35 images per group are plotted relative to those in tissue from uninfected mice with significance determined by one-way ANOVA with Tukey’s post hoc test, *** *p* = 0.0001; **** *p* < 0.0001, ns = not statistically significant.

We also assessed neutrophil infiltration by quantifying Ly6G staining (Figure 7). Mice in the vehicle group challenged with SARS-CoV-2 had increased neutrophils compared with mice in the uninfected group and either dosing of the vaccine reduced neutrophils detected in the lungs of the challenged mice relative to that in the uninfected or vehicle-treated and challenged mice. Collectively, the immune cell infiltration analyses indicated that vaccine-induced protection from severe disease and lethality due to SARS-CoV-2 infection was not due to a dose-dependent difference in immune cell infiltration into the lungs.

### 3.4. Increased CXCL5 Is Correlated with Protection from SARS-CoV-2-Induced Disease and Mortality in K18-hACE2 Mice 

To further elucidate the inflammatory pathways associated with high mortality following SARS-CoV-2 challenge and the impact of vaccination with CoV-RBD121-NP in preventing respiratory disease and pathology, we evaluated cytokines present in post-challenge serum using a 36-plex cytokine panel. By comparing sera from samples taken at day four post-challenge with those obtained at end point, we identified two distinct phases of the infection (early and late), consistent with reports of clinical cases of COVID-19 [29,30,31,32,33,34,35,36,37]. In the early phase of the infection, represented by the sera collected at day four post-challenge, we identified six inflammatory markers that were significantly induced by SARS-CoV-2 in sera from the vehicle group compared with sera from the mice vaccinated with both doses of 45 µg of CoV-RBD121-NP (Table 1). Of the seven inflammatory mediators that increased in the late stage of infection in the mice that received vehicle, three—TNF-α, IL-6, and IP-10—were significantly in both the early and late phases (Table 1). Those that were significantly higher in the vehicle mice at the late stage of infection included the monocyte and neutrophil chemoattractants CCL2, CCL4, and MIP-2a.

We identified a smaller set of cytokines that were different in the sera from the vehicle group and the 45 µg CoV-RBD121-NP group because they were induced in the vaccinated group but either suppressed or only slightly induced in the vehicle group (Table 2). Of these, only CXCL5 was significantly induced in the vaccinated mice at both the early and late stages of infection. These data suggested that CXCL5, in particular, contributes to the protective effect of vaccination.

Inflammatory marker profiling was also performed after SARS-CoV-2 challenge with lung homogenates from mice vaccinated with both doses of 45 µg CoV-RBD121-NP or that received vehicle (Table 3). A similar pattern of changes in inflammatory markers was observed in the lungs as in the sera. Compared with lungs from the vaccinated mice, the lungs from the mice that received vehicle showed an increase in 16 inflammatory markers on the panel of 36 cytokines and seven were also increased in the sera—IL-6, TNF-α, IP-10, CCL2, CCL4, IL-18, and MIP-2a. Collectively, the inflammatory marker profiling of sera and lung tissue indicated that vaccination with the prime and boost dose of 45 µg of CoV-RBD121-NP not only prevents the systemic cytokine storm present in severe COVID-19 but also localized recruitment of inflammatory cells and subsequent damage at the site of infection in the lung.

## 4. Discussion

Vaccination with non-adjuvanted CoV-RBD121-NP at either 15 or 45 µg for both the prime and boost dose induced an antibody response as evidenced by robust neutralization of SARS-CoV-2 in Vero E6 cells and high titers of RBD-directed IgG. The response was dose dependent. In comparison to titers in convalescent human patients, two doses of 15 µg of CoV-RBD121-NP elicited a neutralizing titer of 1261, which was markedly higher than the range observed in human sera from recovered patients (170–960). Increasing the vaccination dose to 45 µg resulted in a 3.14-fold enhancement of the neutralizing titer with a nearly 19-fold increase in detectable RBD-specific antibodies without measurable adverse effects. These data indicated that immunization with CoV-RBD121-NP is an effective strategy for induction of robust neutralizing antibody responses to SARS-CoV-2.

Evaluation of CoV-RBD121-NP in the SARS-CoV-2 challenge model in K18-hACE2 mice demonstrated that immunization with 45 µg of CoV-RBD121-NP provided complete protection from virus-associated lethality and symptomatic disease. The lower dosage of CoV-RBD121-NP (15 µg) provided significant protection from mortality, but only partial protection from symptomatic disease and subsequent lung damage. This symptomatic disease occurred with the lower dosage despite the mice having a pre-challenge neutralizing titer of 1261. In the 45 µg group, SARS-CoV-2 was completely cleared from the lungs and nasal turbinates ten days following challenge. In contrast, two surviving mice in the 15 µg group exhibited low level viral titers in these samples. Histopathological analysis of the lung tissue suggested that, while vaccination at either dosage did not prevent infection with SARS-CoV-2, immunization with 45 µg of CoV-RBD121-NP enabled the mice to neutralize the virus early enough to prevent induction of the pro-inflammatory response and subsequent recruitment of mononuclear cells to the lungs. Our data suggested that the high pre-challenge neutralizing titers detected in the 45 µg group contribute to protection from symptomatic disease.

Based on the correlation between neutralizing titers and protection from symptomatic disease and the low neutralizing titers detected in convalescent patient sera, we propose that viral replication in the respiratory tissue, transmission and breakthrough respiratory symptoms in vaccinated individuals may be the result of an insufficient pre-infection neutralizing antibody titer. Additionally, we found that neutralizing titers in the 15 µg CoV-RBD121-NP group were similar in pre-challenge and day four post-challenge serum samples (Table 1), suggesting that delayed upregulation of B cell antibody production by SARS-CoV-2 results in increased viral replication in partially protected animals. This is particularly notable at the present stage of the pandemic where an increasing number of breakthrough infections are being documented in association with the emergence of the delta variant [15,16,18]. This increase in breakthrough infections is occurring nearly eight months after administration of the first available vaccines and may result from insufficient pre-existing antibody titers produced by an incomplete immune priming strategy (two doses versus three) in an effort to limit adverse effects from vaccines administered following emergency use authorization.

Two distinct phases of COVID-19 have been described [29,30,31,32,33,34,35,36,37]: (1) an early phase with mild to moderate symptoms the first five days of infection with high levels of viral replication prior to the emergence of antibodies and (2) a late phase during which severe symptoms including acute respiratory distress (ARDs), hypercoagulability, and multi-organ failure occur as the result of immune dysregulation and associated tissue damage. Comparison of hematological profiles between patients with relatively mild disease and those with ARDs shows marked elevation in CD14+, IL-1b-producing monocytes in the peripheral circulation [35], infiltration of monocytes and neutrophils into the respiratory tract [35], as well as increased IL-6 and IL-1b in the serum of ARDs patients [30,32,35,36,37]. The high degree of similarity between the clinical presentation and increase in inflammatory markers associated with macrophage activation syndromes, such as hyperferritinemia and disseminated coagulopathy, along with high levels of mononuclear cell infiltrates in the lungs of deceased COVID-19 patients has led to a model in which monocyte and macrophage dysregulation are thought to be responsible for the development of ARDs and mortality in COVID-19 [31,32].

In this study, we examined mice infected with SARS-CoV-2 using a humanized model that represents the severe end of the disease spectrum observed with SARS-CoV-2 infections [27]. By analyzing lung tissue from the infected mice for evidence of neutrophil and monocyte infiltration. we observed marked recruitment of monocytes and neutrophils to the lungs, along with pulmonary edema, necrosis, and hemorrhaging in the vehicle group. These findings are consistent with studies characterizing the K18-hACE2 mouse SARS-CoV-2 challenge model [27,28]. Upon evaluation of sera collected at day four post-challenge and again at end point, we identified an inflammatory marker profile associated with disease and vaccine-induced protection. We observed two distinct phases of inflammation, which mirrors findings for severe human cases of COVID-19 [35,37]. At four days post-challenge, sera collected from the vehicle group contained increased amounts of IFN-γ, IL-6, TNF-α, IL-10, GM-CSF, and IP-10. Vaccination with COV-RBD121-NP (45 µg) significantly reduced all six ‘early’ inflammatory markers and lead to a high level of induction of CXCL5. As infection progressed in the vehicle controls, increased amounts of IL-6 and TNF-α, along with chemokines responsible for monocyte and neutrophil recruitment (CCL2, MIP-2a, and CCL4), were detected in the serum, and this pattern of inflammation was mirrored in the lungs. Vaccination with CoV-RBD121-NP (45 µg) reduced induction of pro-inflammatory cytokines by SARS-CoV-2. Suppression of inflammatory cytokine and chemokines in vaccinated animals correlated with reduction in recruitment of monocytes and neutrophils to the lungs but did not completely eliminate viral-induced pathology in the lungs of the mice that received the 45 µg doses of the vaccine. Taken together, this suggested that the presence of a sufficient pool of neutralizing antibodies may not prevent infection with SARS-CoV-2 but significantly limits the scope of infection and subsequent inflammation-associated lung damage to minimal foci. Furthermore, by restricting SARS-CoV-2 replication, excessive induction of inflammatory cytokines and dysregulation of the immune response were avoided, and viral clearance occurred in vaccinated mice with high levels of neutralizing antibodies.

The observation that CXCL5 was robustly induced in vaccinated mice in this model is notable because CXCL5 has been implicated in the regulation of neutrophil homeostasis and migration in models of *Mycobacterium tuberculosis* (Mtb) infection [38]. CXCL5 inhibits CXCL2 and CXCL1 stimulation of neutrophil activation and extravasation into infected tissues. Depletion of CXCL5 in the context of Mtb infection leads to excessive infiltration of neutrophils into the lungs and subsequent recruitment of monocytes due to elevated cytokine production [38]. The greatest difference in CXCL5 in serum observed in this study occurred at day four following challenge: CXCL5 was markedly reduced in the vehicle group but upregulated in COV-RBD121-NP (45 µg) vaccinated mice. Our data suggested that one of the unidentified early components of the immune response that contributes to monocyte and macrophage dysregulation observed in SARS-CoV-2 infection may be disruption of neutrophil homeostasis through suppression of CXCL5 through an as yet undetermined mechanism. Although we observed the increase in CXCL5 in the vaccinated mice, we cannot determine if this increase related to the RBD or TMV component of the vaccine. Further studies are needed to elucidate the role of neutrophils in the initiation of a macrophage-activation syndrome (MAS)-like condition in mice infected with SARS-CoV-2, as well as the importance of CXCL5 in influencing the outcome of infection.

The CoV-RBD121-NP vaccine candidate is being developed for prophylaxis against COVID-19 disease. Herein, we showed a correlation between neutralizing antibody response and onset of detectable COVID-19 symptoms in the K18-hACE2 mouse model. Our data suggested that high levels of pre-existing neutralizing antibodies may not prevent infection when challenged with high levels of SARS-CoV-2; however, the antibodies curtail symptomatology, inflammation, and lung pathology. Furthermore, these data suggested that neutralizing antibody responses induced by the vaccine support rapid clearance of the virus from lungs and enhance recovery in mice. Overall, our data showed that immunization with CoV-RBD121-NP induces a robust humoral response that subsequently prevents symptomatic disease and virus-associated mortality without evidence of adverse side effects. Evaluation of the doses tested here is currently in phase I clinical trials (NCT04473690). Collectively, the data indicated that such a vaccine has the potential to protect against symptomatic disease caused by SARS-CoV-2 and should continue undergoing clinical evaluation as a COVID-19 vaccine candidate.

## Figures and Tables

**Table 1:** Inflammatory markers induced by SARS-CoV-2 in mice in the vehicle group and suppressed in the mice that received two doses of 45 µg CoV-RBD121-NP. Early represents analysis of pooled sera from mice collected four days post-challenge; late represents analysis of serum from each mouse collected on day 10 or at virus-induced death. Statistical differences were determined by one-way ANOVA with Tukey’s post hoc test. Vehicle group, *n* = 7; CoV-RBD121-NP 45 µg, *n* = 10.

Early			Late		
Cytokine	Fold Reduction 45 µg vs. Vehicle	*p* Value	Cytokine	Fold Reduction 45 µg vs. Vehicle	*p* Value
IL-10 IFN-γ IP-10 GM-CSF TNF-α IL-6	2.58 2.38 2.18 2.13 1.93 1.25	0.0011 <0.0001 0.0206 <0.0001 <0.0001 0.0008	TNF-α IL-6 IL-18 IP-10 CCL2 CCL4 MIP-2a	78.62 16.84 11.18 4.16 5.40 2.83 1.43	0.0273 0.0176 0.0053 <0.0001 0.0161 0.0002 0.036

**Table 2:** Inflammatory markers suppressed or minimally induced by SARS-CoV-2 in mice in the vehicle group and induced in mice that received two doses of 45 µg CoV-RBD121-NP. Early represents analysis of pooled sera from mice collected four days post-challenge; late represents analysis of serum from each mouse collected on day 10 or at virus-induced death. Statistical differences were determined by one-way ANOVA with Tukey’s post hoc test. Vehicle group, *n* = 7; CoV-RBD121-NP 45 µg, *n* = 10.

Early			Late		
Cytokine	Fold Increase 45 µg vs. Vehicle	*p* Value	Cytokine	Fold Increase 45 µg vs. Vehicle	*p* Value
CXCL5 IL-1a IL-12p70	40.92 2.54 2.11	<0.0001 <0.0001 0.0025	CXCL5	6.57	0.0044

**Table 3:** Differences in inflammatory markers in lung homogenates from mice in the vehicle and 45 µg CoV-RBD121-NP groups after SARS-CoV-2 challenge at death or experiment end. Statistical differences were determined by one-way ANOVA with Tukey’s post hoc test. Vehicle group, *n* = 7; CoV-RBD121-NP 45 µg, *n* = 10.

Inflammatory Marker	Fold Change Vehicle vs. 45 µg	*p* Value
MCP-3	90.73	<0.0001
IL-6	66.79	0.0453
CCL2	20.86	0.0022
IP-10	18.26	<0.0001
IL-12p70	9.36	0.0084
IL-18	8.90	0.002
CCL3	7.21	0.0074
TNF-α	6.00	0.0001
CCL4	5.40	0.0041
IL-10	3.66	0.0004
GM-CSF	3.18	0.0056
MIP-2a	3.04	<0.0001
IL-13	2.73	0.0141
IL-27	1.74	0.0011
IL-22	1.62	0.0008
IL-15	1.41	0.0007

## Data Availability

The data is contained within the article.
